# Editorial: Molecular and cellular bases of peripheral neuropathies

**DOI:** 10.3389/fnmol.2024.1423363

**Published:** 2024-05-13

**Authors:** Jorge Feito, Jose Antonio Vega

**Affiliations:** ^1^Servicio de Anatomía Patológica, Instituto de Investigación Biomédica de Salamanca, Complejo Asistencial Universitario de Salamanca, Salamanca, Spain; ^2^Departamento de Morfología y Biología Celular, Universidad de Oviedo, Oviedo, Spain; ^3^Facultad de Ciencias de la Salud, Universidad Autónoma de Chile, Santiago de Chile, Chile

**Keywords:** peripheral nerve injury, peripheral neuropathies, neuroregeneration, neuropathic pain, gut microbiota, animal models, molecular pathways, mechanotransduction

As the editors of this Research Topic, we are pleased to present the different articles that, altogether, constitute an advance in the knowledge of the peripheral neuropathies. The Research Topic was primarily focused in a better understanding of these pathologies, including the molecular mechanisms underlying them. Daifallah et al. reviewed the small fiber neuropathy (SNF), suggesting that pathogenic antibodies contribute to this entity. Chen et al. reviewed the chemotheraphy-induced peripheral neuropathy (CIPN), including the pathogenesis, diagnosis and, particularly, the molecular mechanisms underlying the development of the disease: cytoskeletal disruption, mitochondrial disfunction, damage in dorsal root ganglia (DRG) neurons or neuroinflammation.

This objective was not limited to particular diseases, and also included general bases of pathology, such as demyelination or injury responses, such as regeneration. The first point is included in the research by Chernov and Shubayev about the sexual dimorphism in degenerating peripheral nerves and found estrogens and estrogen receptors as sex-dependent regulators of injury response, mostly downregulating differentially expressed genes; this RNA-sequencing study also found increased interleukin-6 expression and lipocalins of the major urinary protein in males. The second point about regeneration of injured peripheral nerve was addressed by Li et al., who demonstrated the boosting effect of nerve fibroblasts in nerve regeneration compared to cardiac fibroblasts, thanks to Activin A expression. Moreover, a voltage-gated calcium channel subunit α2δ-1 was found by Koga et al. to enhance excitatory synaptic transmission and mechanical hypersensitivity after peripheral nerve injury in spinal dorsal horn neurons.

Papers concerning pain sensation were highly regarded. A great example of this is found in the paper by Garcia-Mesa et al. about blood vessels in diabetic neuropathy. Although the increase in blood vessel density is already known regarding diabetic peripheral neuropathy (Stirban, 2014), this paper demonstrates the relationship between painful diabetic neuropathy and the Piezo2 expression. Piezo2 is the second of the mechanotransductory Piezo channels, and is particularly tuned to mechanosensation in contrast with the more polymodal Piezo1 (García-Mesa et al., 2017). The role of pain in SNF was also detailed in the paper by Daifallah et al.. Although more focused in the therapeutic options, the mechanisms of peripheral neuropathic pain were reviewed in the article by Pacifico et al., including the pathogenesis of different neuropathies.

Research regarding the enteric nervous system was also welcomed. This aspect of the peripheral nervous system is incorporated in the review of the CIPN, which specifically covers the influence of gut microbiota in the enteric nervous system in this pathology (Chen et al.). This relation between gut microbiota and peripheral neuropathies, in this case with cancer-related pain, was found by Yuan et al.. These authors found significant potentiation of the morphine effect by reshaping gut microbiota in rats.

The second objective was related with the improvement of the therapeutic options regarding these neuropathies. In this line, the treatment of CIPN was reviewed by Chen et al.. Regarding pain treatment, Yuan et al. proposed probiotics supplementation to improve the control of cancer pain. Furthermore, Koga et al., in their research of excitatory SDH neurons, obtained results that help to better understand the pharmacology of gabapentinoids.

In addition, therapy of neuropathic pain was widely reviewed by Pacifico et al. exploring new and experimental agents targeted against specific mutations or mechanisms like neurotrophins and their receptors. These authors particularly highlight potential therapeutic interventions, like targeting DRG neurons, axonal degeneration or cutaneous nociceptors; we found particularly elegant their review about cutaneous interactions in peripheral neuropathic pain.

## Author contributions

JF: Conceptualization, Writing—original draft. JV: Writing—review & editing.
